# Amphiphilic Lignin Nanoparticles Made from Lignin-Acrylic Acid-Methyl Methacrylate Copolymers

**DOI:** 10.3390/nano12152612

**Published:** 2022-07-29

**Authors:** Yingchao Wang, Niloofar Alipoormazandarani, Lauren Skye Puumala, Weijue Gao, Shanshan Liu, Fangong Kong, Qiang Wang, Pedram Fatehi

**Affiliations:** 1 State Key Laboratory of Biobased Material and Green Papermaking, Qilu University of Technology (Shandong Academy of Sciences), Jinan 250353, China; wyc19940530@126.com (Y.W.); liushanshan@qlu.edu.cn (S.L.); kfg@qlu.edu.cn (F.K.); 2 Green Processes Research Centre, Lakehead University, 955 Oliver Road, Thunder Bay, ON P7B 5E1, Canada; nalipoor@lakeheadu.ca (N.A.); lauren.puumala@alumni.ubc.ca (L.S.P.); wgao@lakeheadu.ca (W.G.)

**Keywords:** lignin, acrylic acid, methyl methacrylate, copolymerization, amphiphilic, nanoparticles

## Abstract

In this study, a novel amphiphilic KL-AA-MMA nanoparticle was prepared through the graft copolymerization of kraft lignin (KL) with acrylic acid (AA) and methyl methacrylate (MMA), using potassium persulfate as an initiator in a water/dimethyl sulfoxide solvent medium, which was followed by the nanoprecipitation technique using dimethylformamide as a solvent and deionized water as an antisolvent. The successful graft polymerization was verified by ^1^H-nuclear magnetic resonance (NMR), ^31^P-NMR, and Fourier transform infrared (FTIR) analyses; and the grafting yield of the generated KL-AA-MMA copolymer ranged from 68.2% to 96.5%. Transmission electron microscopy (TEM) observation revealed the formation of amorphous KL-AA-MMA nanoparticles. Additionally, KL-AA-MMA9 nanoparticles with the highest yield exhibited the minimum hydrodynamic diameter and polydispersity of 261 nm and 0.153, respectively. Moreover, the amphiphilicity of KL-AA-MMA nanoparticles was significantly improved by the grafting of MMA monomers. Finally, the adsorption performance of KL-AA-MMA nanoparticles at the xylene interface was evaluated by a quartz crystal microbalance with dissipation (QCM-D). The results demonstrated that the most amphiphilic sample, KL-AA-MMA9 nanoparticles, with the smallest hydrodynamic size displayed the highest adsorption on the oil/water interface. This product provides a wide range of applications in oil/water emulsions.

## 1. Introduction

Lignin is a renewable natural polymer composed of phenylpropane units linked by ether bonds and is a significant byproduct of the pulp and paper industry [1,2]. Kraft lignin (KL) is a lignin derivative originating from the kraft pulping process that has become a representative of industrial lignin due to its significant production rate and relatively low production costs [3,4]. However, only about 2% of the 50 million tons/year of generated KL is used to create heat and replace fossil materials in pulp mills [5]. Thus, KL is still considered an underutilized resource [6]. Recently, KL has been used for the preparation of various high-value-added functional materials—including lignin nanoparticles (LNP)—because of its rich availability, renewable source, and unique physicochemical properties [7,8].

As a nanomaterial with abundant surface-active groups, LNP has gained extensive attention in the materials community due to the combined advantages of its non-toxicity, sustainability, renewability, and widespread presence in nature [9]. In recent years, LNP has been widely used as a Picking emulsion stabilizer due to its green and affordable features, good dispersibility, stability, and oxidation resistance [10]. Nevertheless, lignin nanoparticles generated from the original KL possessed a series of shortcomings, such as weak hydrophilicity, which severely restricted their practical applications. Fortunately, the polymerization of KL can effectively overcome these issues [11,12,13]. At present, many technologies in the polymerization of lignin have been developed, including ring-opening [14], atom transfer radical polymerization [15], and free radical polymerization [3,16]. Among them, free radical graft copolymerization is an ideal method for producing hydrophobic KL derivatives [17]. In various monomers grafted onto KL, methyl methacrylate (MMA) can provide attractive characteristics in KL-MMA products, such as hydrophobicity, elasticity, and thermal resistance [13,18]. In contrast, acrylic acid (AA) monomers can endow KL-AA with outstanding hydrophilic performance [12,19]. For emulsion stabilizers, improved lignin amphiphilicity is critical, and can be enhanced by the copolymerization of KL with AA and MMA. In this respect, the application of KL-AA-MMA polymers in the preparation of hydrophilic–lipophilic nanoparticles has never been studied, and so is the main objective of this study.

Herein, we report on novel amphiphilic KL-AA-MMA nanoparticles that were manufactured by the graft copolymerization of KL with AA and MMA monomers in a potassium persulfate initiator system, followed by a nanoprecipitation technique. FTIR and ^1^H-NMR verified the success of the copolymerization reaction, and the phenolic hydroxyl group content of the KL-AA-MMA polymer was calculated by quantitative ^31^P-NMR analysis. Nanoparticle formation was visualized by TEM imaging and the hydrodynamic diameter and polydispersity of various KL-AA-MMA nanoparticles are discussed. Additionally, the amphiphilicity of KL-AA-MMA nanoparticles on solid surfaces was assessed by their two- (water) and three-phase (oil) contact angles. Finally, the adsorption performance of KL-AA-MMA nanoparticles at the xylene interface was studied using a quartz crystal microbalance with dissipation (QCM-D) experiment.

## 2. Experiments

### 2.1. Materials

Softwood kraft lignin (KL) was obtained from FPInnovations in Thunder Bay, ON, Canada, and was produced via LignoForce^TM^ technology. Analytical grades of acrylic acid (AA), methyl methacrylate (MMA), potassium persulfate (KPS), dimethyl sulfoxide (DMSO), dimethylformamide (DMF), sodium hydroxide (NaOH), hydrochloric acid (HCl), acetone, deuterated dimethyl sulfoxide ([D_6_]DMSO), deuterium oxide (D_2_O), deuterated chloroform (CDCl_3_), 2-chloro-4,4,5,5-tetramethyl-1,2,3-dioxapospholane, anhydrous pyridine, cyclohexanol, chromium (III) acetylacetonate, sodium dodecyl sulfate (SDS), and silica (SiO_2_) were all purchased from the Sigma-Aldrich Company, Oakville, Ont. Canada. Xylene (≥98.5%, ACS grade as a mixture containing ortho, meta, and para isomers) was bought from Fisher Scientific, Toronto, Ont. Canada. The SiO_2_-coated piezoelectric Q-Sense sensor (QSX 303) was supplied by Q-Sense, Biolin Company, Gothenburg, Sweden. Cellulose acetate dialysis membranes with a molecule weight cut off of 1000 g/mol were received from Spectrum Labs. Inc., New Brubswick, NJ, USA. All chemicals were used without further purification. HPLC-grade Milli-Q water was used in the QCM-D studies and deionized water was used in the other experiments.

### 2.2. Copolymerization of KL with AA and MMA

First, 1 g of KL was dissolved in 20 mL of a 1:1 mixture of water and DMSO by stirring at 200 rpm for 5 min in a 250 mL three-neck flask. Second, 6 mL of AA was mixed with 10 mL of deionized water and added to the flask under stirring. Then, the reaction mixture was adjusted to pH 3 using an aqueous NaOH solution. Next, the desired amount of MMA (0, 3, 6, or 9 mL) was added to the flask, which was followed by transferring the flask to a preheated water bath on a hot plate at 80 °C under stirring at 280 rpm. Subsequently, 5 mL of a 6 g/L KPS solution was added to the reaction mixture of water: DMSO (1:1, *v*/*v*), with a total volume of 50 mL. Reactions were performed at 80 °C for 3 h. Meanwhile, the flask was equipped with a condenser, and the reaction mixture was purged with nitrogen throughout the reaction process.

After completing the reaction, the reaction contents were cooled by immersing the flask in cool running water. Next, 100 mL of deionized water was added to the flask under stirring at 280 rpm. The mixture was acidified to pH 2 using HCl. Then, 10 mL of acetone was added to the mixture, and the mixture was further acidified to pH 1.5 with HCl. Afterward, the acidified solution was added to centrifuge tubes, and an extra 5 mL of deionized water was added to each tube. Then, the tubes were centrifuged at 4000 rpm for 10 min. Following centrifugation, all sediments were collected in beakers, and then, the pH of the collected pastes was adjusted to 7 using an aqueous NaOH solution. The products were purified by dialysis against deionized water for 48 h, and the water was replaced every 6 h. Lastly, the purified KL-AA and KL-AA-MMA solutions were dried in an oven at 105 °C overnight. The grafting yield of the product was calculated according to Equation (1) [13]:

Grafting yield (%) = (W_2_ − W_1_)/W_1_ × 100% (1)

where W_1_ (g) is the initial weight of the KL used in the reaction and W_2_ (g) is the total solid weight obtained after purification.

### 2.3. Characterization of KL-AA and KL-AA-MMA Copolymers

The successful copolymerization of KL with AA and MMA was evaluated by quantitative ^31^P-NMR, according to a previous method [20]. Briefly, 35 mg of dried sample was dissolved in 500 μL of anhydrous pyridine/CDCl_3_ mixture (1.6:1, *v*/*v*). Then, 200 μL of cyclohexanol (21.48 mg/mL) was added as the internal standard and 50 μL of chromium (III) acetylacetonate solution (5.6 mg/mL) was added to the pyridine/CDCl_3_ solution as the relaxation reagent. Finally, 100 μL of phosphitylating reagent II (2-chloro-4,4,5,5-tetramethyl-1,2,3-dioxapospholane) was added and transferred into a 5 mm NMR tube for NMR acquisition. The ^31^P-NMR spectra of the KL, KL-AA, and KL-AA-MMA samples were recorded using an INOVA-500 MHz NMR spectrometer, Varian, (Peabody, MA, USA) with a Quad probe dedicated to ^31^P.

^1^H-NMR spectroscopy analyses of the KL, KL-AA, and KL-AA-MMA samples were performed using the INOVA NMR spectrometer operating at 500 MHz with a 45° pulse and a relaxation delay time of 1.0 s. Before testing, all dried samples were dissolved in [D_6_]DMSO and D_2_O and then stirred for 30 min for ^1^H-NMR measurements.

The functional groups of the dried KL, KL-AA, and KL-AA-MMA samples were determined by a Fourier transform infrared (FTIR) spectrometer (Bruker, Tensor 37, Karlsruhe, Germany) at 25 °C using a universal attenuated-total-reflection (ATR) probe in the wavenumber range of 4000–600 cm^−1^, with a resolution of 4 cm^−1^.

The charge density of the KL-AA and KL-AA-MMA copolymers was measured by a particle charge detector, Mütek PCD 04 titrator (Arzbergerstrae, Herrsching, Germany), with a 0.005 mol/L of PDADMAC solution. Each sample was measured in triplicate and the average values were recorded.

### 2.4. KL-AA-MMA Nanoparticle Formation

KL-AA-MMA nanoparticles were formed by the nanoprecipitation technique using DMF as a solvent and deionized water as an antisolvent. Briefly, 1 mg/mL of KL-AA and KL-AA-MMA solutions were prepared in DMF by stirring for 12 h at 200 rpm and 40 °C. Then, the solutions were filtered with 0.45 μm disposable syringe filters. Afterward, the filtered solutions were dialyzed with deionized water in a dialysis membrane for 48 h, with the water being replaced every 6 h. Finally, various KL-AA-MMA nanoparticle suspensions were generated.

### 2.5. TEM Imaging Analysis of KL-AA-MMA Nanoparticles

The morphologies of KL-AA-MMA nanoparticles were observed using a JEM-2100Plus transmission electron microscopy (TEM, JEOL, Tokyo, Japan) with an accelerating voltage of 100 kV. All TEM samples were prepared by depositing one drop (5 μL) of the dialyzed nanoparticle solutions onto the carbon-coated copper grid of the TEM instrument and then dried naturally at 25 °C before imaging. TEM imaging was captured without a stain in the bright field mode with a slight under-focusing.

### 2.6. Hydrodynamic Diameter Determination of KL-AA-MMA Nanoparticles

A dynamic light scattering instrument (DLS, BI-200SM Brookhaven Instruments Corp., Uptown, NY, USA) was used for measuring the hydrodynamic diameter of the KL-AA-MMA nanoparticles. The light source was a solid-state laser with a maximum power of 35 mW and a wavelength of 637 nm. The analysis was conducted at 25 ± 0.02 °C and the scattering angle was set at 90 °. Each sample was measured for 2 min, and the reported result in this paper is the average of three repetitions.

### 2.7. Contact Angle Measurement of KL-AA-MMA Nanoparticles

The water (WCA) and oil (OCA) contact angles for the KL-AA and KL-AA-MMA nanoparticle-coated surfaces at the air/water and oil/water interfaces were determined by a Theta Lite optical tensiometer (Biolin Scientific, TL100, Stockholm, Swedem) that was equipped with Attension software using a sessile drop method. In this set of experiments, the solid surfaces were first prepared by coating the KL-AA or KL-AA-MMA nanoparticle suspensions (3 μL, 0.5 wt%) onto clean glass slides using a spin coater (WS-400B-NPP) spin-processor (Laurell Technologies Corp, North Wales, PA, USA) at 1500 rpm for 20 s in a nitrogen atmosphere, and the wet films were dried at 80 °C for 12 h [21]. This procedure was repeated 9 more times to form a total of ten layers of nanoparticles on the glass slides. Then, 4 μL of deionized water was placed on the nanoparticle-coated glass slides within 10 s using a micro-syringe, and then the WCA was measured at room temperature. Afterward, the OCA was determined by carefully transferring the above slide into a glass chamber filled with xylene (a mixture containing ortho, meta, and para isomers). The sessile drop method was used for measuring the three-phase contact angle at 25 °C for 10 s.

### 2.8. QCM-D Studies of KL-AA-MMA Nanoparticles

The adsorption performance of the KL-AA and KL-AA-MMA nanoparticles at the xylene/water interface was assessed using a quartz crystal microbalance with dissipation monitoring (QCM-D, 401 E1 Q-Sense AB, Gothenburg, Sweden). In this study, we chose a SiO_2_-coated piezoelectric Q-Sense sensor as the substrate for the xylene coating, since previous studies have reported effective interactions between the two materials [22]. Before measurements, the sensors were cleaned by treating them in a UV/ozone oxidation cleaner (PSD Series, digital UV ozone system) for 15 min, followed by immersion in 2 wt% SDS solution for 2 h [23]. Subsequently, the sensors were rinsed thoroughly with Milli-Q water and then dried with nitrogen gas. Next, 5 μL of xylene was spin-coated onto the sensors at 3000 rpm for 30 s with an acceleration of 200 m/s^2^ under an N_2_ atmosphere and then dried in an oven at 80 °C for 10 min [24]. This spin-coating and drying process was repeated 9 times to produce 10 coats of xylene on the sensors.

QCM-D adsorption experiments were conducted on the xylene-coated SiO_2_ sensors by first introducing Milli-Q water buffer to the flow chamber of the instrument for longer than 5 min to establish a stable baseline. Then, KL-AA and KL-AA-MMA nanoparticles (50 mg/L) at pH 4.5 were introduced into the flow chamber with a flow rate of 0.15 mL/min at 20 °C, and frequency and dissipation changes were monitored for 8 min. The fundamental frequency of the crystal sensor and the representative data were recorded from the 3rd overtone. After that, the sensors were rinsed with Milli-Q water for 3.5 min to monitor desorption. A temperature of 20 °C and a pump rate of 0.15 mL/min were set throughout all the QCM-D experiments [25]. The curves of the resonance frequency (Δf) and dissipation factor (ΔD) were recorded accordingly over 15 min. Film thickness and mass modeling were simulated using the Q-Tools software. The Voigt viscoelastic model was selected for modeling the results by conducting a numerical fit to the frequency and dissipation data for overtones of 3. In this study, D was larger than 1 × 10^−6^ per every 10 Hz frequency change, indicating that the evaluation process based on the Voight model of viscoelasticity had to be used [26]. The Sauerbrey equation only considers frequency changes for calculating an adsorbed layer’s thickness, which is only valid for rigid films [26]. The fluid viscosity and density were assumed to be 1.05 mPa·s and 0.99 g/cm^3^, respectively [21].

## 3. Results and Discussion

### 3.1. Synthetic Process of KL-AA-MMA Nanoparticles

**Figure 1:**
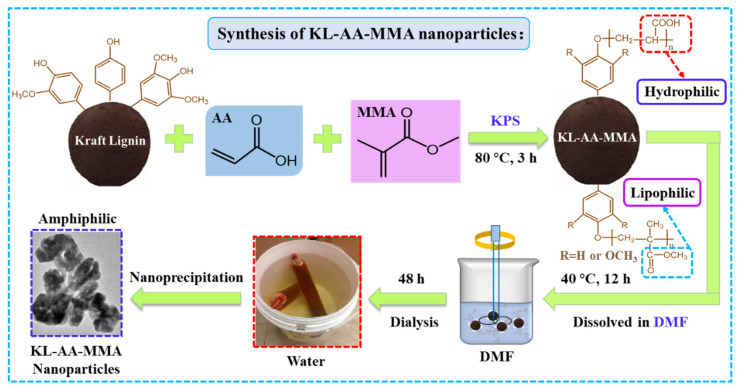
Schematic illustration for the synthetic process of KL-AA-MMA nanoparticles.

In recent years, lignin nanoparticles have attracted great attention—particularly in the field of oil/water stabilizers. This is attributed to their unique merits, such as their acceptable dispersibility, long-term stability, good biocompatibility, and anti-oxidation activity [27]. However, the traditional lignin nanoparticles produced from unmodified lignin still suffer from the defects of weak hydrophobicity and a lack of hydrophilicity, which would severely hamper their practical application. To address this challenge, amphiphilic KL-AA-MMA nanoparticles were fabricated via free-radical polymerization combined with nanoprecipitation technology in this study, and the synthetic process was illustrated in Figure 1. Briefly, a KL-AA-MMA copolymer was prepared via a copolymerization reaction of KL with AA and MMA in the water/DMSO solvent medium using KPS as an initiator. In this system, the presence of AA endowed the product with excellent hydrophilicity, while the grafting of MMA imparted outstanding hydrophobicity and chain extension. Moreover, the results confirmed that the grafting yield of the KL-AA-MMA copolymer varied between 68.2 and 96.5% (Table 1). Meanwhile, the grafting ratio increased by increasing the ratio of MMA. Subsequently, the amphiphilic KL-AA-MMA nanoparticles were produced from this copolymer through a solvent exchange using DMF as a solvent and deionized water as an antisolvent.

### 3.2. Copolymerization Verification of KL with AA and MMA

**Figure 2:**
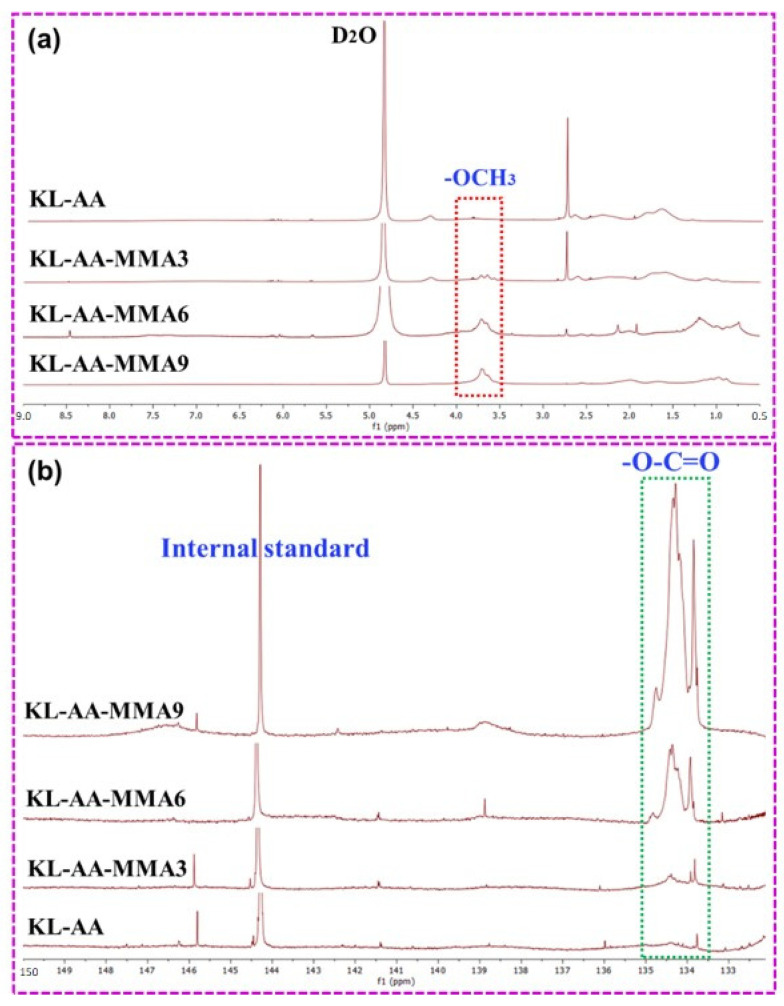
(**a**) ^1^H-NMR and (**b**) ^31^P-NMR spectra of the KL-AA and KL-AA-MMA copolymers.

To verify the successful copolymerization of KL with AA and MMA, the chemical structure of KL-AA and KL-AA-MMA samples was determined by ^1^H-NMR and ^31^P-NMR. As seen in Figure 2a, a new resonance at 3.5–4 ppm emerged for all KL-AA-MMA copolymers, which was assigned to the methoxy group (-OCH_3_) of MMA [28]. The appearance of the remarkable chemical shift in the KL-AA-MMA compared to the KL-AA in their ^1^H-NMR spectra confirmed the grafting of MMA to the KL skeleton. The intensity for the -OCH_3_ peak of the KL-AA-MMA9 was significantly greater than that of the KL-AA-MMA3, which indicated that the grafting ratio of the KL-AA-MMA9 was higher than that of the KL-AA-MMA3. This result corresponded to the data in Table 1. Furthermore, the presence of an AA monomer on the KL backbone was attested by the ^31^P-NMR spectra. Figure 2b shows a carboxyl (-COOH) correlated with the AA and an ester carbonyl (-O-C=O) originating from the MMA at 135.5–134.2 ppm [29], which authenticates the success of the copolymerization reaction.

To better investigate the copolymerization efficiency of KL with AA and MMA, the hydroxyl group contents of the KL-AA and KL-AA-MMA copolymers were calculated by quantitative ^31^P-NMR analysis [30]; the results are listed in Table 2. The KL-AA polymer contained 1.26 mmol/g of aliphatic OH and 2.16 mmol/g of phenolic OH. After copolymerization with the MMA monomer, the phenolic OH levels in the KL-AA-MMA copolymer gradually reduced. Additionally, the phenolic hydroxyl content was reducedmore greatly for the KL-AA-MMA with higher MMA levels; this decrease was ascribed to the occupation of this reactive site upon AA and MMA copolymerization [13]. The lowest content of phenolic OH in the KL-AA-MMA9 sample was 0.5 mmol/g, illustrating that most phenolic hydroxyl groups in the original KL participated in the copolymerization reaction. Moreover, the carboxylic OH content was raised significantly from 0.49 mmol/g (KL-AA) to 4.01 mmol/g (KL-AA-MMA9)—such an augment reflects an eightfold improvement in carboxyl content (Table 2). These results demonstrated that the grafting ratio of the KL-AA-MMA9 was the highest, which supports the results reported by the ^1^H-NMR.

**Figure 3:**
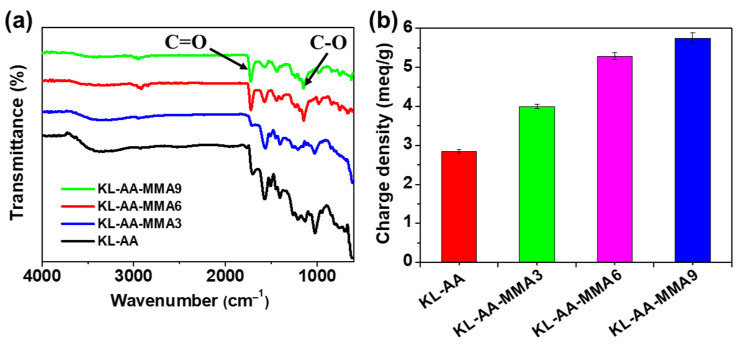
(**a**) FTIR spectra (4000–600 cm−^1^) and (**b**) charge density of KL-AA and KL-AA-MMA copolymers.

Additionally, the FTIR spectra confirmed that AA and MMA monomer chains were successfully grafted onto the KL backbone. Figure 3a displays the aromatic skeletal vibration of KL in the region of 1450–1600 cm^−1^ [31]. Additionally, the characteristic peak at around 3400 cm^−1^ was interpreted as the aliphatic or aromatic hydroxy groups of lignin [32]. Compared with the other copolymers, the KL-AA-MMA9 copolymer exhibited the highest intensity peaks at 1731 cm^−1^ (O-C=O) and 1143 cm^−1^ (C-O) [33], which further proved the successful polymerization of KL with AA and MMA. In addition, the carboxylic acid groups derived from AA were introduced into the KL molecules during the copolymerization process, thus increasing the charge density of the obtained KL-AA-MMA copolymer (Figure 3b).

### 3.3. Morphology, Size, and Contact Angle Analyses of KL-AA-MMA Nanoparticles

**Figure 4:**
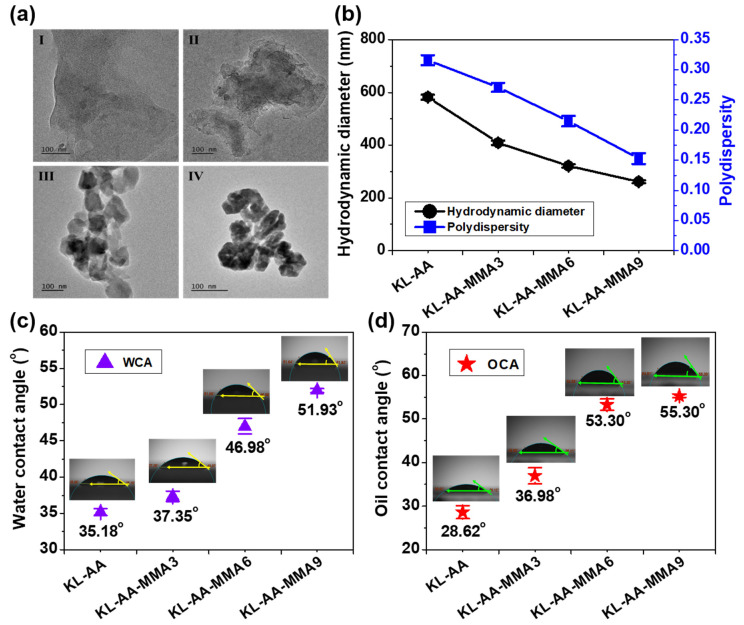
(**a**) TEM images of (**I**) KL-AA, (**II**) KL-AA-MMA3, (**III**) KL-AA-MMA6, and (**IV**) KL-AA-MMA9 nanoparticles. (**b**) Hydrodynamic diameter and polydispersity, (**c**) water and (**d**) oil contact angles of various KL-AA-MMA nanoparticles.

The microscopic morphologies of various KL-AA-MMA nanoparticles were observed by TEM, as presented in Figure 4a. It was found that the KL-AA polymer did not form nanoparticles in the nanoprecipitation process (Figure 4aI), while amorphous nanoparticles of KL-AA-MMA were visualized (Figure 4aIV). This is because the polymerization of water-soluble AA made the KL-AA sample have an inferior self-assembly ability with hydrophilic features. In contrast, the grafting of MMA endued the KL-AA-MMA copolymer with hydrophobic groups—so, the KL-AA-MMA sample engendered superior attractive interactions [34,35]. Meanwhile, the higher the grafting yield of the KL-AA-MMA nanoparticles, the smaller the nano-size would be. The KL-AA-MMA9 nanoparticles with the highest yield exhibited the smallest particles, with a diameter of 264 ± 8.6 nm. Moreover, the hydrodynamic diameter (R_h_) and polydispersity of the KL-AA-MMA nanoparticles were further investigated, and the data are revealed in Figure 4b (additional data is available in Figure S1 in supplementary material). The average R_h_ and polydispersity of the nanoparticles were reduced from 408 nm and 0.271 in the KL-AA-MMA3 to 261 nm and 0.153 in the KL-AA-MMA9, respectively, which is in agreement with the TEM images. However, since R_h_ determines the hydrodynamic diameter of KL-AA-MMA nanoparticles in solution, but TEM displays the size of nanoparticles in the dry state, the size of the particles was different when observed by these techniques. In general, drying the polymeric precipitate affects the morphology and dimension of nanoparticles [36].

In addition, to predict the adsorption performance of KL-AA-MMA nanoparticles at the xylene interface, the two- (water, WCA) and three-phase (oil, OCA) contact angles for the KL-AA-MMA nanoparticle surfaces at the air/water and oil/water interfaces were measured, respectively [37]. WCA was employed to define the wettability of the KL-AA-MMA nanoparticle droplets on a glass surface. The WCA and OCA values of the KL-AA-MMA nanoparticle surfaces are described in Figure 4c,d. It can be seen that the WCA value on the surface of the KL-AA sample without polymerized MMA was the lowest at 35.18 ± 0.48 ° and that the coated surface was instantly wetted by water droplets (inset in Figure 4c). This implies that the KL-AA sample was hydrophilic, with the worst compatibility toward the oil interface. MMA polymerization resulted in a gradual increase in the WCA and OCA of the KL-AA-MMA nanoparticle surfaces, which was remarkably increased to 51.93 ± 0.29 ° and 55.3 ± 0.39 ° for the KL-AA-MMA9 nanoparticles with the largest grafting yield, respectively (Figure 4c,d). This phenomenon confirmed that the grafting of MMA was conductive to the amphiphilic behavior of KL-AA-MMA nanoparticles. The KL-AA-MMA9 nanoparticles demonstrated the highest compatibility with the oil interface, contributing to the subsequent adsorption at the xylene interface.

### 3.4. QCM-D Adsorption Performance of KL-AA-MMA Nanoparticles

**Figure 5:**
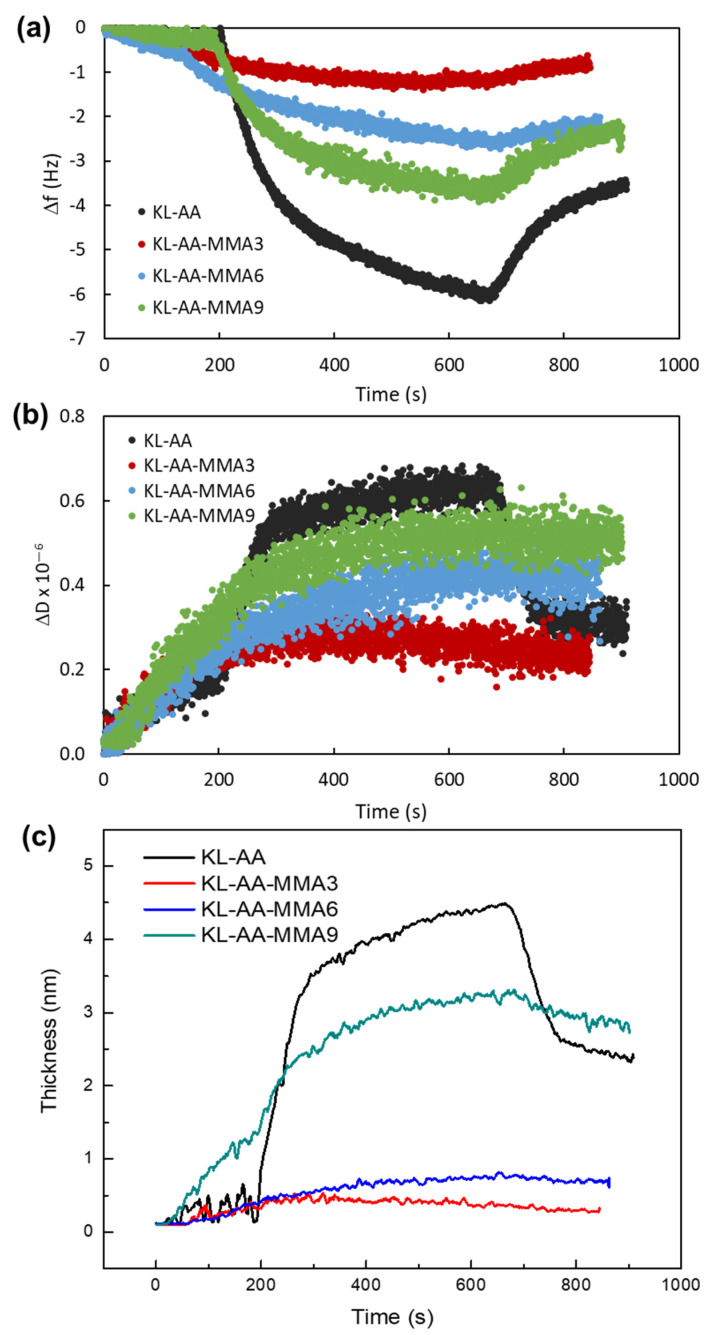
(**a**) Frequency and (**b**) dissipation at the third overtone for the adsorption of KL-AA and KL-AA-MMA nanoparticles on xylene-coated SiO_2_ sensors. (**c**) thickness growth over time for KL-AA and KL-AA-MMA nanoparticles obtained via the Voigt model.

The adsorption performance of the KL-AA-MMA nanoparticles at the xylene interface was assessed in detail by a quartz crystal microbalance with dissipation (QCM-D). The frequency (Δf) and dissipation (ΔD) in Figure 5a,b show the adsorption affinity and viscoelasticity of the adlayer at the xylene-coated surface, respectively. Comparing the KL-AA-MMA nanoparticles, the most amphiphilic sample—KL-AA-MMA9, with the smallest hydrodynamic size—displayed the largest change in frequency compared to the KL-AA-MMA3 and KL-AA-MMA6, indicating that the amount of adlayer mass adsorbed onto the xylene-coated surface increased with the increasing amphiphilicity of KL-AA-MMA. The dissipation followed a similar pattern, and the KL-AA-MMA9 had a higher ΔD than the KL-AA-MMA3 and KL-AA-MMA6 (Figure 5b)—which suggests that the viscoelasticity of the KL-AA-MMA adlayers increased with increases in its amphiphilicity. The Voigt model was implemented to further investigate the adsorption process by comparing the thickness growth of the adlayer as a function of time (Figure 5c). The KL-AA-MMA9 adsorbed more than other KL-AA-MMA nanoparticles and displayed the highest thickness. KL-AA showed larger changes in Δf and ΔD and a higher thickness than all KL-AA-MMA nanoparticles, which was probably attributable to its having the highest hydrophilicity levels, leading to the adsorption of more water (i.e., swelling) in the KL-AA adlayer as QCM revealed the adsorption of the total mass, i.e., the polymer and water [38]. After rinsing with water, the adsorbed thickness of KL-AA significantly dropped, indicating the desorption of loosely attached KL-AA from the xylene-coated surface. However, most adsorbed KL-AA-MMA adlayers remained attached, suggesting that the KL-AA-MMAs were firmly bound to the xylene/water interface. These results indicated that amphiphilic KL-AA-MMA nanoparticles can adhere to an oil surface and have the potential to be applied for oil/water emulsion stabilization.

## 4. Conclusions

In this work, the grafting of kraft lignin (KL) with acrylic acid (AA) and methyl methacrylate (MMA) was carried out via simple free-radical polymerization in the presence of potassium persulfate as an initiator. The chemical structure of KL before and after graft copolymerization was investigated by ^1^H-NMR, ^31^P-NMR, and FTIR analyses. The increased methoxy (-OCH_3_) and ester carbonyl (-O-C=O) contents and the decreased phenolic hydroxyl contents confirmed that AA and MMA monomers were successfully grafted onto the KL backbone. Additionally, the grafting yield of the resulting KL-AA-MMA product ranged from 68.2% to 96.5%. The nanoparticle production of the generated lignin copolymers proved that the inclusion of MMA facilitated the generation of nanoparticles, which was ascribed to both the hydrophilic and hydrophobic features of the KL-AA-MMA copolymer. Additionally, MMA inclusion resulted in a gradual increase in the water and oil contact angles of the KL-AA-MMA nanoparticles, which were raised to 51.9° and 55.3° for the KL-AA-MMA9 nanoparticles with the largest grafting yield—demonstrating that the KL-AA-MMA9 nanoparticles possessed excellent amphiphilic behavior. TEM imaging verified the formation of KL-AA-MMA nanoparticles.

Furthermore, the hydrodynamic size and polydispersity of nanoparticles were reduced from 408 nm and 0.271 in the KL-AA-MMA3 to 261 nm and 0.153 in the KL-AA-MMA9, as the proportion of MMA increased in the copolymer, respectively. Additionally, the quartz crystal microbalance with dissipation (QCM-D) experiment indicated that KL-AA-MMA9 nanoparticles showed the highest adsorption on the xylene/water interface owing to its having the highest amphiphilicity and the smallest hydrodynamic size. This product can potentially serve as an oil/water stabilizer for application in various industrial fields.

## 5. Application and Future Studies

Due to its relatively weak interfacial activity, a tremendous amount of lignin is untapped in industrial applications and acts as a byproduct of the pulping industry. Thanks to the grafting of AA and MMA to lignin, an amphiphilic KL-AA-MMA polymer was produced, which was beneficial for the formation of nanoparticles. The potential application of the modified lignin nanoparticle is as a Pickering emulsion stabilizer, which could be used in mixtures of water and hydrophobic liquids. Unlike traditional nonionic or ionic surfactant-based emulsion, Pickering emulsion is stabilized by solid particles, and can form an interface between the dispersed and continuous phases. In future studies, the effect of KL-AA-MMA nanoparticles on the stability of various oil–water Pickering emulsions will be studied.

## Figures and Tables

**Table 1:** Reaction conditions and grafting yields of KL-AA and KL-AA-MMA copolymers.

Sample Label	Water/DMSO (*v*/*v*)	AA (mL)	MMA (mL)	KPS (mL)	Temperature (°C)	Time (h)	Grafting Yield (%)
KL-AA	1/1	6	0	5	80	3	68.2
KL-AA-MMA3	1/1	6	3	5	80	3	86.9
KL-AA-MMA6	1/1	6	6	5	80	3	91.0
KL-AA-MMA9	1/1	6	9	5	80	3	96.5

**Table 2:** Hydroxyl group contents of the KL-AA and KL-AA-MMA copolymers calculated from ^31^P-NMR spectra (Figure 2).

Chemical Shift	Assignment	Content (mmol/g)			
		KL-AA	KL-AA-MMA3	KL-AA-MMA6	KL-AA-MMA9
149.0–145.0	Aliphatic OH	1.26	1.10	0.93	0.43
135.5–132.8	Carboxylic OH	0.49	0.90	2.43	4.01
143.2–140.5	Syringyl OH	0.92	0.81	0.44	0.18
140.0–137.9	Guaiacyl OH	0.70	0.62	0.45	0.30
137.7–136.3	p-Hydroxyl-phenyl OH	0.54	0.44	0.21	0.02
Total phenolic OH		2.16	1.87	1.10	0.50

## Data Availability

Raw data will be available upn requoest from the corresponding authors.

## Supplementary Materials

The following supporting information can be downloaded at: https://www.mdpi.com/article/10.3390/nano12152612/s1, Figure S1: Hydrodynamic size distribution of different KL-AA-MMA nanoparticles.
