# Supplementary material for: Amphiphilic Lignin Nanoparticles Made from Lignin-Acrylic Acid-Methyl Methacrylate Copolymers
Source: Nanomaterials (Basel). 2022 Jul 29;12(15):2612. doi: 10.3390/nano12152612 (PMC9370363; doi:10.3390/nano12152612)
Supplement: Supplementary file 1 [file nanomaterials-12-02612-s001.zip › nanomaterials-1834623-supplementary.pdf]

nanomaterials

Article

Amphiphilic Lignin Nanoparticles made from Lignin-Acrylic Acid-Methyl Methacrylate Copolymers

Yingchao Wang [1,2], Niloofar Alipoormazandarani [2], Lauren Skye Puumala [2], Weijue Gao [2], Shanshan Liu [1], Fangong Kong [1], Qiang Wang [1,*] and Pedram Fatehi [1,2,*]

[1] State Key Laboratory of Biobased Material and Green Papermaking, Qilu University of Technology (Shandong Academy of Sciences), Jinan 250353, China; wyc19940530@126.com (Y.W.); liushanshan@qlu.edu.cn (S.L.); kfg@qlu.edu.cn (F.K.)

[2] Green Processes Research Centre, Lakehead University, 955 Oliver Road, Thunder Bay, ON P7B 5E1, Canada; nalipoor@lakeheadu.ca (N.A.); lauren.puumala@alumni.ubc.ca (L.S.P.); wgao@lakeheadu.ca (W.G.)

* Correspondence: wangqiang83@qlu.edu.cn (Q.W.); pfatehi@lakeheadu.ca (P.F.); Tel.: +1 (807)-343-8697

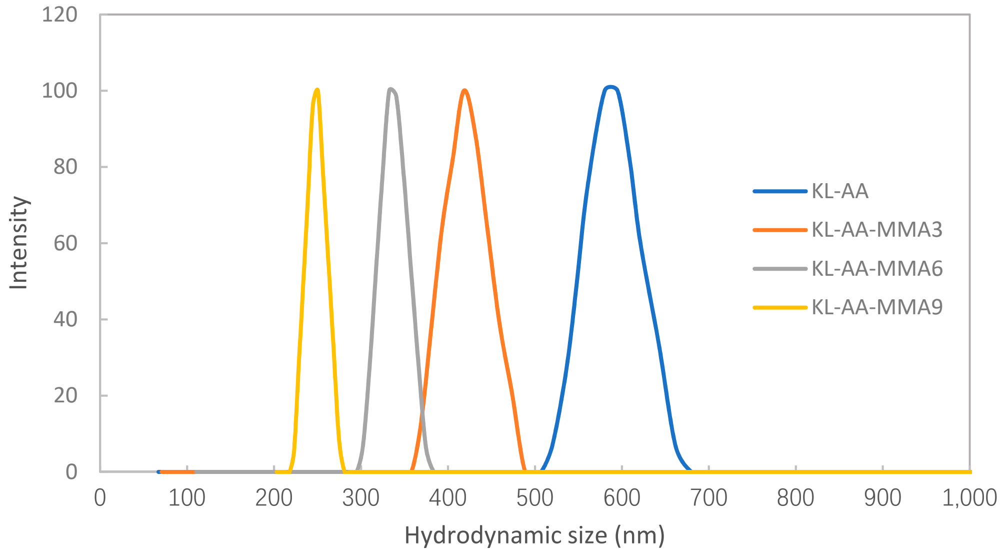

Figure S1. Hydrodynamic size distribution of different KL-AA-MMA nanoparticles.
